# The Expression of Inflammatory Mediators in Bladder Pain Syndrome

**DOI:** 10.1016/j.eururo.2016.02.058

**Published:** 2016-08

**Authors:** Ifeoma Offiah, Athanasios Didangelos, John Dawes, Rufus Cartwright, Vik Khullar, Elizabeth J. Bradbury, Suzanne O'Sullivan, Dic Williams, Iain P. Chessell, Kenny Pallas, Gerry Graham, Barry A. O’Reilly, Stephen B. McMahon

**Affiliations:** a Neurorestoration Group, Wolfson Centre for Age Related Diseases, King's College London, London, UK; b Department of Epidemiology and Biostatistics, Imperial College London, London, UK; c Department of Urogynaecology, Cork University Maternity Hospital, University College Cork, Wilton, Co. Cork, Ireland; d Neuroscience IMED, MedImmune, Cambridge, UK; e The Beatson Institute for Cancer Research, Glasgow, UK

**Keywords:** Animal model, Bladder pain syndrome, CCL21, Clinical correlation, FGF7, Gene expression analysis, Interstitial cystitis, Pain behaviour

## Abstract

**Background:**

Bladder pain syndrome (BPS) pathology is poorly understood. Treatment strategies are empirical, with limited efficacy, and affected patients have diminished quality of life.

**Objective:**

We examined the hypothesis that inflammatory mediators within the bladder contribute to BPS pathology.

**Design, setting, and participants:**

Fifteen women with BPS and 15 women with stress urinary incontinence without bladder pain were recruited from Cork University Maternity Hospital from October 2011 to October 2012. During cystoscopy, 5-mm bladder biopsies were taken and processed for gene expression analysis. The effect of the identified genes was tested in laboratory animals.

**Outcome measures and statistical analysis:**

We studied the expression of 96 inflammation-related genes in diseased and healthy bladders. We measured the correlation between genes and patient clinical profiles using the Pearson correlation coefficient.

**Results and limitations:**

Analysis revealed 15 differentially expressed genes, confirmed in a replication study. *FGF7* and *CCL21* correlated significantly with clinical outcomes. Intravesical *CCL21* instillation in rats caused increased bladder excitability and increased *c-fos* activity in spinal cord neurons. *CCL21* atypical receptor knockout mice showed significantly more *c-fos* upon bladder stimulation with *CCL21* than wild-type littermates. There was no change in *FGF7*-treated animals. The variability in patient samples presented as the main limitation. We used principal component analysis to identify similarities within the patient group.

**Conclusions:**

Our study identified two biologically relevant inflammatory mediators in BPS and demonstrated an increase in nociceptive signalling with *CCL21.* Manipulation of this ligand is a potential new therapeutic strategy for BPS.

**Patient summary:**

We compared gene expression in bladder biopsies of patients with bladder pain syndrome (BPS) and controls without pain and identified two genes that were increased in BPS patients and correlated with clinical profiles. We tested the effect of these genes in laboratory animals, confirming their role in bladder pain. Manipulating these genes in BPS is a potential treatment strategy.

## Take Home Message

Bladder pain syndrome (BPS) pathology remains poorly understood, and its treatment is difficult. We identified mediators that, from our study of their action in the bladders of laboratory animals, may be implicated in disease pathology. These mediators are unexploited therapeutic targets for BPS treatment.

## 1 Introduction

Bladder pain syndrome (BPS) is pain related to the urinary bladder accompanied by frequency, urgency, or nocturia, with the exclusion of any other diseases of the lower urinary tract [1], [2]. Although the disease affects both sexes, women are more commonly affected than men by 5:1 [3]. Some patients display a mild form of the disease, with treatment generally orchestrated in the outpatient setting; in other cases, the disease is debilitating and requires prolonged hospitalisation and, often, repeated surgical intervention.

The aetiology of BPS is unknown. Multiple theories exist, including epithelial disruption and mast cell and vascular abnormalities [4], [5]. The contributions of peripheral neuronal mechanisms remain unclear. We explore the hypothesis that many patients have a peripheral inflammatory disorder and that the expression of inflammatory mediators in the bladder wall activates and sensitises the bladder sensory afferents, driving BPS symptoms. Because there are currently no disease-modifying treatments for BPS, we postulated that the identification of novel inflammatory mediators associated with the disease might be manipulated to alter the disease's course.

We used quantitative gene expression analysis of 96 inflammatory mediators to measure gene expression levels in BPS and control samples. We then tested the activity of the identified mediators in an animal model of BPS using the enzymes chondroitinase and heparanase to digest the proteoglycan barrier [6], [7].

## 2 Materials and methods

We performed a prospective observational study of 15 women with BPS and 15 age-matched female controls between October 2011 and October 2012 at Cork University Maternity Hospital. BPS participants had bladder pain for at least 3 mo, with urodynamic and cystoscopic evidence of disease. Controls were patients undergoing tension-free vaginal tape surgery. Patients who had systemic disease such as malignancy, coagulopathies, or other forms of cystitis (eg, infective, chemical, or radiation cystitis) were excluded.

Participants completed the O’Leary-Sant Interstitial Cystitis Symptom and Problem Index (ICS/PI) questionnaire. While under general anaesthesia, a rigid cystoscopy (30° lens) was performed, and three 5-mm biopsies were taken from above the bladder trigone by cold-cup biopsy technique from each participant [7]. Biopsies were also taken from healthy-looking bladder away from lesion sites. RNA was extracted from the tissue using a combination of phenol extraction and column purification. RNA integrity was determined using an Agilent RNA 6000 Bioanalyzer (Agilent Technologies, Waldbronn, Germany).

We performed reverse transcription reactions using a complementary DNA reverse transcription kit (Invitrogen; Thermo Fisher Scientific, Waltham, MA, USA). Custom-made TaqMan microfluidic cards (Applied Biosystems; Thermo Fisher Scientific, Waltham, MA, USA) were used to measure expression levels of 96 inflammatory mediators in disease versus control tissue calculated using the ΔCT (cycle time) method [8] and normalised against the geometric mean of three housekeeping genes using the ReadqPCR and NormqPCR packages [9].

### 2.1 Patient clustering

We used principal component analysis with eigenvalue decomposition to visualise biologic variability within the patient group [10]. We identified patient clusters based on their gene expression levels and used the first two components for repeat gene expression analysis. Pearson correlation coefficient was used to visualise the hierarchical clustering of each group of patients for significantly dysregulated genes.

### 2.2 Replication study

Confirmatory gene expression analysis was replicated in an independent cohort of 23 patients with bladder pain and 15 controls without pain. BPS was diagnosed based on history of bladder pain, and control participants were patients who had lower urinary tract disorders such as stress urinary incontinence or overactive bladder without pain. Biopsies were taken under general anaesthesia from the bladder dome away from lesion sites using the cold-cup biopsy technique.

### 2.3 Statistical analysis

Data analysis was performed using the ΔCT method [8]. We applied the Benjamini-Hochberg false discovery rate (FDR) algorithm (5%) to the data. We used a volcano plot to illustrate variation within the data, thus visually highlighting differentially regulated genes (*t* test *p* value = 0.01; twofold difference). Spearman rank correlation was used to determine the correlation of gene expression levels against BPS clinical phenotypes derived from the ICS/PI questionnaire.

### 2.4 Animal experiments

All experiments were conducted using adult female Wistar rats (approximate weight: 200–250 g, Harlan, UK) in accordance with the UK Home Office Regulations. All rats were housed in the licensed biological services unit of King's College London with a 12-h day/night cycle. Food and water were available at all times. Each animal was randomly assigned to treatment protocols, and assessors were blinded to the treatments the rats received.

### 2.5 Cystometry

Fifteen animals were anaesthetised with 1 mg/kg urethane; 20-gauge catheters were inserted transurethrally and attached to a syringe pump and pressure transducer. Bladders were distended with 0.9% saline, with 50 μl/min and baseline cystometric analysis recorded. Ten animals had 200 μl of 0.25 IU chondroitinase ABC and heparanase III (Sigma-Aldrich, St. Louis, MO, USA) bladder instillation. Five control animals had 200 μl phosphate-buffered saline (PBS) instilled, and solutions were allowed to remain in situ for 2 h. Then, five animals each had 10 μl of 250 ng/ml of either *CCL21* or *FGF7* instilled for 2 h. Cystometric analysis was repeated, and total contraction time was measured.

### 2.6 Behavioural assessment

Pelvic pain response was assessed using calibrated Von Frey monofilaments. Von Frey withdrawal is typically performed on the hind paw of laboratory animals. We performed mechanical withdrawal assessment on the suprapubic region, which is reported as a valid method for the assessment of referred hyperalgesia and mechanical allodynia in animal models of bladder hypersensitivity [11]. Tactile sensitivity of the suprapubic region was assessed using the Chaplan method [12]. A positive behavioural response was recorded as licking or scratching of the stimulated area, sharp withdrawal, or jumping. Rats were anaesthetised with isoflurane and transurethrally catheterised; 10 rat bladders were permeabilised with 200 μl of 0.25 IU chondroitinase ABC and heparanase III. Experimental rats received 10 μl of 250 ng/ml *CCL21* or *FGF7* postdigestion (five per group), while five controls received PBS. Von Frey assessment was repeated, and 50% threshold values were calculated.

### 2.7 Spinal c-fos expression

Fifteen animals were anaesthetised with urethane, transurethrally catheterised, and treated with 200 μl of 0.25 IU chondroitinase ABC and heparanase III. Then, five animals each had 10 μl of 250 ng/ml of either *CCL21* or *FGF7* instilled. Five control animals were catheterised without bladder instillation. After 2 h, all animals were sacrificed and transcardially perfused with 4% paraformaldehyde (VWR, Lutterworth, UK). Spinal cord sections L6–S1 were collected, cryoprotected, embedded, and frozen. Serial (20 μm) sections were cut and stained for *c-fos.* In brief, sections were incubated for 48 h in a 1:1000 dilution of rabbit *c-fos* antibody (Cell Signalling Technology, Danvers, MA, USA), with a 1:500 dilution of mouse monoclonal anti-Neu N antibody (Merck Millipore, Darmstadt, Germany) in 10% normal donkey serum. Sections were washed with PBS, then incubated in secondary antibodies of donkey antimouse Alexa Fluor 488 (Thermo Fisher Scientific) and donkey antirabbit Alexa Fluor 546 (1:500; Thermo Fisher Scientific) for 2 h. Sections were washed and mounted with VECTASHIELD Antifade Mounting Medium with DAPI (Vector Laboratories, Burlingame, CA, USA). Images were taken using a ZEISS LSM 710 confocal microscope (Oberkochen, Germany). Cells exhibiting *c-fos* immunoreactivity were counted in the afferent regions of the spinal cord.

### 2.8 CCL21 atypical receptor knockouts

We received 17 C57/BL6/J mice from The Beatson Institute for Cancer Research, Glasgow: 9 ACKR4 −/− receptor knockout and 8 wild-type littermates [13]. The mice were anaesthetised with urethane, catheterised, and bladder permeabilised using 150 μl of 0.25 IU chondroitinase ABC and heparanase III. Then, 10 μl of 250 ng/ml *CCL21* was instilled for 2 h, following which all animals were sacrificed, perfused, fixed, and spinal *c-fos* staining performed.

## 3 Results

BPS participants had urodynamic and cystoscopic evidence of disease. Control participants had no urologic disease or pain symptoms. All participants were free from urinary tract infections. See supplementary material for further phenotypic details.

**Fig. 1:**
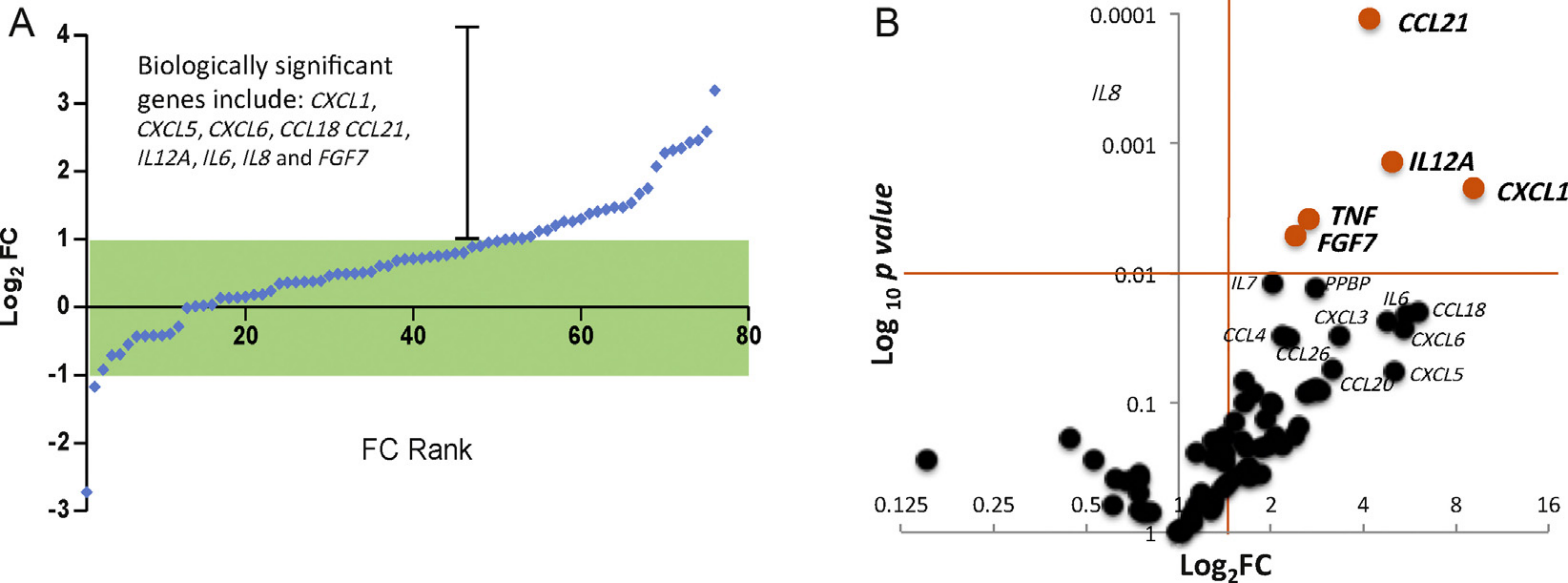
Gene expression analysis of all patients. (A) Gene expression levels in bladder biopsies of bladder pain syndrome (BPS) patients versus controls. Each point represents the mean fold change (FC) for each gene transcript, displayed as the log_2_ of the FC. Each point is ranked in order of FC, commencing with the genes with the lowest FC to those that are most upregulated (compared to controls). The green box denotes genes with FC ±2. FC ranking revealed that a considerable number of genes were upregulated in the data set. (B) Volcano plot visualising the genes separated according to their expression FC (*x*-axis) and significance (*y*-axis: log_10_*p* value) in BPS and controls. Genes highlighted in red had log_2_ FC >2 and log_10_*p* values <0.01 in our data set. FC = fold change.

Gene expression analysis of 96 inflammation-related genes revealed 15 differentially regulated genes (fold change >2; *p* < 0.05), highlighting a clear inflammatory process in the BPS pathology (Fig. 1A). The volcano plot shows the five most dysregulated genes (*CCL21, IL12A, CXCL1, TNF*, and *FGF7* [shown in red]), which were all significantly dysregulated after correcting *p* values using the FDR method of Benjamini-Hochberg, with a 5% FDR.

### 3.1 Gene correlation with clinical phenotypes

**Fig. 2:**
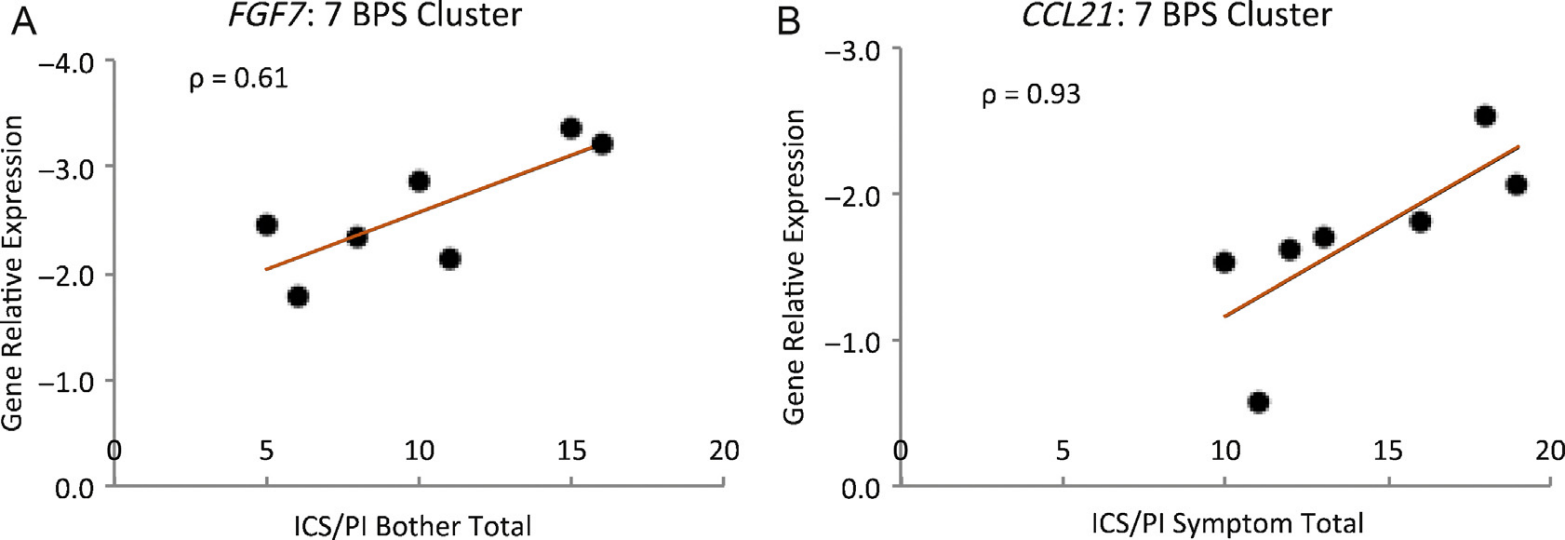
Correlation of gene relative expression with patient clinical phenotypes. Spearman rank correlation coefficient of the seven bladder pain syndrome patients, selected using principal component analysis, with the clinical phenotypes. There was a positive correlation between transcript expression for *CCL21* and *FGF7* and the O’Leary-Sant Interstitial Cystitis Symptom and Problem Index questionnaire scores (*CCL21* ρ = 0.93, *p* = 0.0067; *FGF7:* ρ = 0.61, *p* = 0.17). BPS = bladder pain syndrome; ICS/PI = O’Leary-Sant Interstitial Cystitis Symptom and Problem Index.

Spearman rank correlation was used to compute correlation between the dysregulated genes and patient clinical phenotypes; it revealed that *CCL21* and *FGF7* were positively correlated with patient clinical phenotypes for ICS/PI symptom and problem indices, respectively (Fig. 2). In the case of *CCL21*, the correlation was statistically significant.

### 3.2 Principal component analysis

**Fig. 3:**
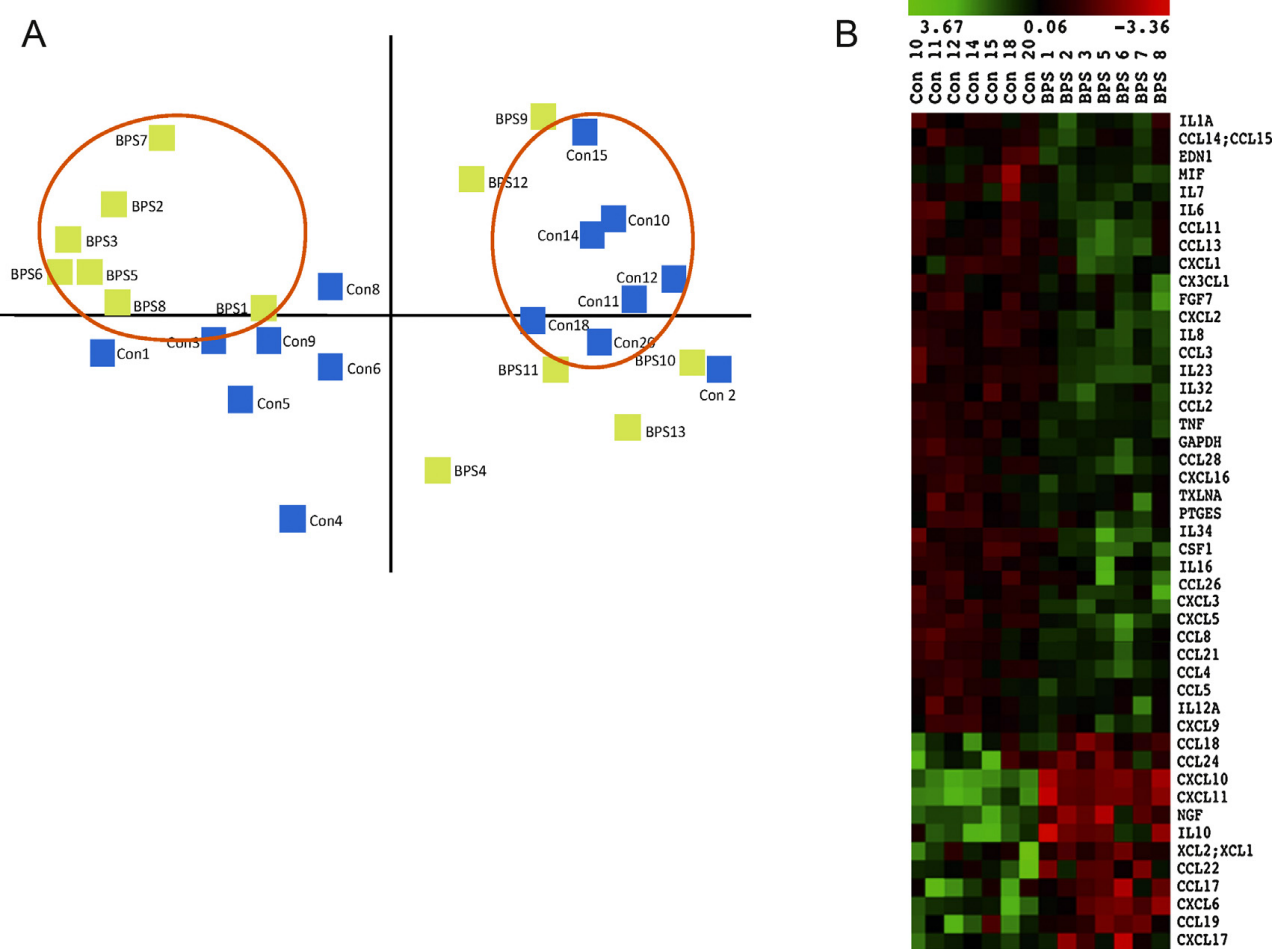
Principal component analysis and hierarchical clustering. (A) Principal component analysis of all patients analysed, evaluated based on their gene expression profiles. The first two principal components, consisting of two clusters of seven bladder pain syndrome (BPS) patients and seven controls, are circled in red. (B) Hierarchical clustering of differentially regulated genes of the seven BPS and seven control patients identified with the principal component analysis. Green denotes upregulated genes, whereas red denotes downregulated genes. BPS = bladder pain syndrome.

We applied principal component analysis to the entire gene expression data set (15 BPS and 15 controls) to visualise variability in the data. Figure 3A shows the first two dimensions of this analysis, from which we identify a cluster of seven BPS and seven controls that were tightly grouped. Further analysis of these principal component analysis–selected cohorts by hierarchical clustering revealed, as expected, a striking reproducibility among patients for all differentially regulated genes (Fig. 3B). A ΔCT analysis of this refined group revealed 35 significantly dysregulated inflammatory genes, including *CCL21* and *FGF7*.

### 3.3 Replication study

**Fig. 4:**
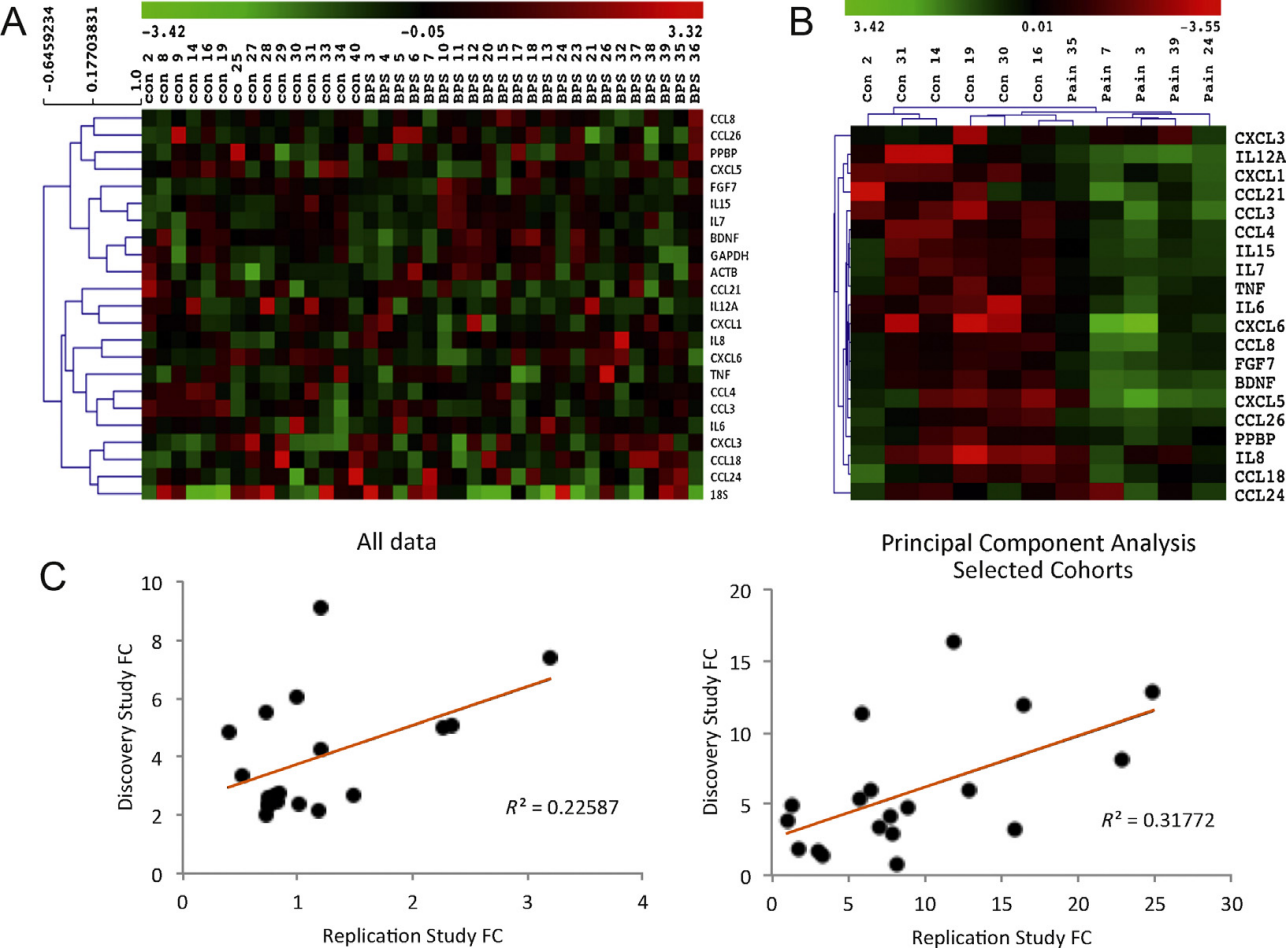
Replication study and correlation with primary study. (A) Hierarchical clustering of all participants, showing large biologic variability. (B) Hierarchical clustering of the principal component analysis–selected first two components, showing the correlation between patients and genes. (C) Each point represents the fold change (FC) difference for each gene from the discovery and replication study. The left panel corresponds to the correlation of the FCs for all patients included in both analyses (*R^2^* = 0.23, *p* = 0.03). The second panel represents the seven bladder pain syndrome versus seven controls from the initial study, correlated with the five pain patients and six controls from the replication study (*R^2^* = 0.32; *p* = 0.01). Significance was calculated using Pearson *R* with a 2-tailed *t* test and a 95% confidence interval. FC = fold change.

We performed a transcriptional analysis on an independent cohort of patients. In this cohort, hierarchical clustering revealed large biologic variability (Fig. 4A), but principal component analysis revealed a cluster of 5 pain and 6 controls, ΔCT analysis of which showed significant dysregulation of 15 inflammatory genes, including again *CCL21* and *FGF7* (Fig. 4B). This gene dysregulation pattern for both the original cohort and the principal component analysis–selected cluster correlated with that of the primary study (Fig. 4C).

### 3.4 Preclinical studies

#### 3.4.1 CCL21 increases bladder contractions and pain-related behaviour

**Fig. 5:**
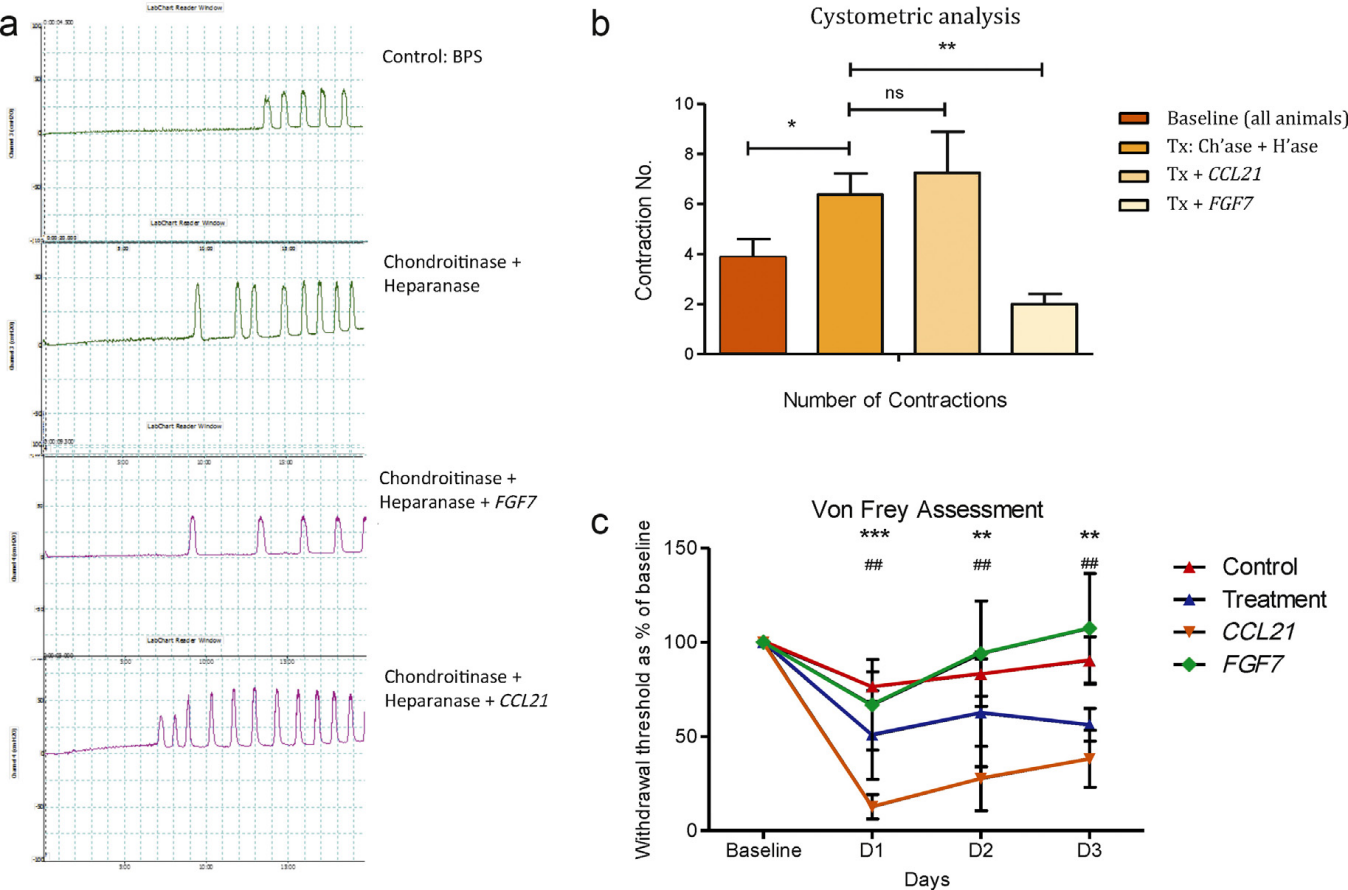
Cystometry and behaviour assessment. (A) Representative cystometric recordings of bladders, showing contraction frequency following baseline assessment, enzymatic proteoglycan deglycosylation, *FGF7* treatment, and *CCL21* treatment in permeabilised bladders. (B) Quantification of the cystometric number of bladder contractions. Displayed is the mean total number of contractions in a 20-min interval ±1 standard error of the mean (SEM; five rats per group). Statistical significance calculated using one-way analysis of variance (ANOVA) with Bonferroni post-test correction. (C) Behavioural assessment. Each point represents the mean mechanical withdrawal threshold as a percentage of baseline ±1 SEM (five rats per group). Statistical significance was calculated using two-way ANOVA with Bonferroni correction. The *CCL21* treatment withdrawal threshold is significantly lower than the control group and animals’ postenzymatic deglycosylation alone. BPS = bladder pain syndrome; Ch’ase = chondroitinase; H’ase = heparanase; ns = not significant; Tx = treatment. (B) * *p* < 0.05; ** *p* < 0.01. (C) * Significance difference in *CCL21* from baseline: *** *p* < 0.001; ** *p* < 0.01; ^#^ significance difference in treatment from baseline: ^##^*p* < 0.01.

To assess the effect of *CCL21* and *FGF7* in laboratory animals, we permeabilised the bladders of female Wistar rats using chondroitinase ABC and heparanase III to digest proteoglycans of the mucosal barrier, then applied the ligands intravesically. Cystometric analysis revealed an increase in bladder contraction number following enzymatic deglycosylation, which was sustained but not additive after *CCL21* treatment (*p* = 0.0067). There was no change from baseline following *FGF7* treatment (*p* = 0.320) (Fig. 5A and 5B). In addition, behavioural pain assessment showed a significant decrease in the mechanical withdrawal threshold following deglycosylation, with withdrawal thresholds further decreasing following *CCL21* treatment. There was no difference in withdrawal threshold in the *FGF7* or control rats (Fig. 5C).

#### 3.4.2 CCL21 leads to upregulation of spinal c-fos

**Fig. 6:**
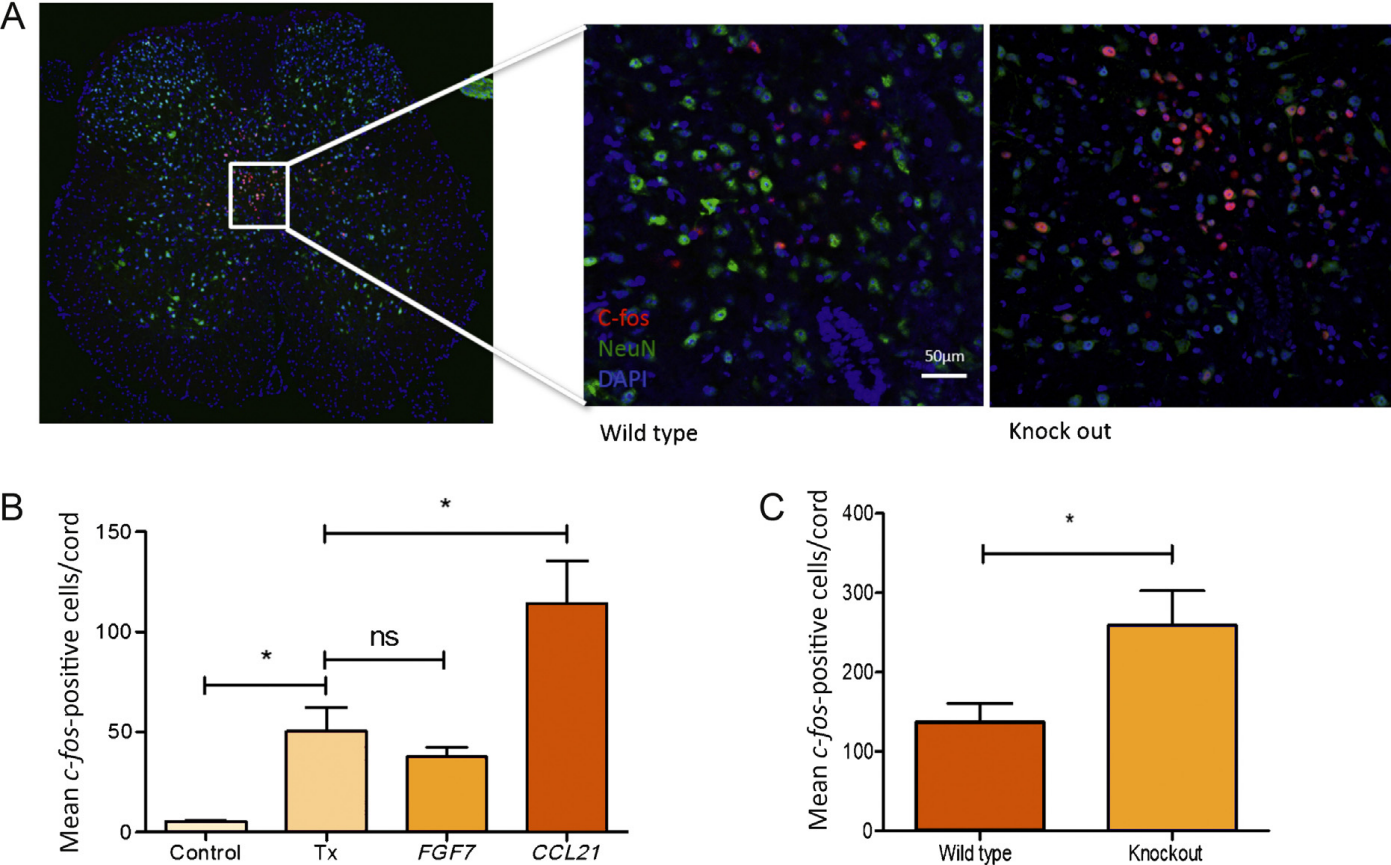
The effect of ligand treatment on *c-fos*–positive cell number. (A) Representative image of a spinal cord with *c-fos* staining plus high-power magnification of the dorsal commissure showing *c-fos* positivity in a wild-type and knockout mouse. (B) Quantification of *c-fos*–positive cell numbers in rats. Displayed is the mean number of *c-fos*–positive cells in the L6–S1 spinal cord (five rats per group). Statistical significance was calculated with one-way analysis of variance with Bonferroni post-test correction. (C) Quantification of *c-fos*–positive cell numbers in *CCL21* atypical receptor knockout mice and wild-type littermates. Displayed is the mean number of *c-fos*–positive cells in the L6–S1 spinal cord (six wild type, seven knockout). Statistical significance of *p* = 0.039 was calculated with an unpaired Student *t* test. ns = not significant; Tx = treatment. * *p* < 0.05.

The effect of the ligands on spinal neurone activation was assessed by immunolocalisation of the immediate-early gene, *c-fos*, in anaesthetised rats following bladder permeabilisation. Analysis revealed a significant increase in the *c-fos*–positive cell number following urothelial permeabilisation compared to the saline-treated controls (*p* = 0.036). There was a further significant increase following *CCL21* treatment (*p* = 0.042). There was no significant difference between the animals after permeabilisation alone compared to those after *FGF7* treatment (*p* = 0.27) (Fig. 6B).

To assess the role of the *CCL21* atypical chemokine receptor 4 (ACKR4) on bladder pain, we performed immunolocalisation of *c-fos* on ACKR4 knockout mice and wild-type littermates. Assessment revealed a significant increase in the number of *c-fos*–positive cells in the ACKR4 knockout mice compared to the wild-type controls (Fig. 6A and 6C).

## 4 Discussion

Our study shows upregulation of specific genes encoding inflammatory mediators in bladder biopsies of BPS patients. We also showed a significant correlation between mRNA levels of the genes *FGF7* and *CCL21* with patient clinical profiles and disease severity scores. In addition, we highlighted the role of *CCL21* as a pain mediator in an animal model of BPS.

In the past 20 yr, various animal models of cystitis have been created [14]. These models rely on inflammation of the bladder urothelium and so replicate only part of the pathology of the human disease. We have disrupted the bladder barrier by specific deglycosylation of urothelial proteoglycan molecules, thus replicating the disease, because 80% of BPS patient bladder biopsies show features of a defective barrier [15].

The key problem with human analysis is biologic variability, especially with complex diseases like BPS. In our study, principal component analysis revealed clear biologic variability in the participants analysed, but it also identified a cluster of patients who had distinct transcriptional profiles, suggesting that although a substantial number of patients share common inflammatory pathology, this does not apply to all patients. We therefore used this method to examine the biologic variability of the replication study, identifying a cluster with a strongly described inflammatory phenotype. The replication study was not designed to be a formal biologic replicate. The recruitment criteria for that study were slightly different and less exhaustive than the original one, but we took the opportunity to replicate the analysis in a similar cohort of patients with bladder pain.

The modest number of participants represents the main limitation of our study. This is a consequence of the difficulty of recruiting BPS patients resulting from the inherent diagnostic complexity of this syndrome. This sample size is, however, similar to previous studies in publication evaluating gene expression profiles of bladder biopsies from BPS patients as well as a quantitative analysis of gene expression, which, although small, were able to detect differences [16], [17], [18], [19].

There is great heterogeneity of findings in the field of molecular characterisation of BPS. Results vary because of the complexity of the disease, the difficulty of diagnosis, the and phenotypic variability in BPS patients. We have confirmed an inflammatory component to the disease process. Several candidate genes that were found upregulated in our study have been reported as significant in BPS, including *NOS, IL6, IL8, IL10, IL17A, NGF*, and *TNFA* [16], [20], [21]. Of these, we identified two genes that correlated to patient phenotypes: *FGF7* and *CCL21. FGF7*, also known as *keratinocyte growth factor*, expression is found to be upregulated in chronically injured tissue [22]. *FGF7* expression is associated with healing and wound repair, supporting the integrity of the gastrointestinal tract mucosal barrier in chemotherapy patients who have oral mucositis [23], [24]. It is possible that upregulation of *FGF7* has a similar action in BPS patient bladders, strengthening urothelial barrier integrity. Our results corroborate these findings; *FGF7* treatment following urothelial permeabilisation reduced bladder excitation on cystometry and behaviour analysis. In addition, *FGF7* treatment did not lead to an increase in *c-fos*–positive cell numbers, suggesting that *FGF7* may be involved in repair of the damaged urothelium.

*CCL21*, also known as *secondary lymphoid tissue chemokine*, is a potent inflammatory chemokine that regulates dendritic cell migration and has been implicated in various chronic inflammatory, fibrotic, and pain conditions, including rheumatoid arthritis, neuropathic pain, idiopathic pulmonary fibrosis, chronic hepatitis C, and primary biliary cirrhosis [25], [26], [27], [28]. In the central nervous system, *CCL21* expression is increased in damaged neurons, leading to increased microglial P2X4 expression [26]. It is possible that *CCL21* may be involved in similar inflammatory cell activation in the periphery in response to injury. We have shown that *CCL21* activity leads to an increase in bladder excitability and pain. The atypical *CCL21* receptor ACKR4, found in the epithelial cells of the heart, thymus, skin, and urinary bladder, is a high-affinity receptor for *CCL21* [29]. By internalising its ligands, this receptor diminishes the available circulating *CCL21* levels, suppressing disease severity [13]. In our study, the increased number of spinal cells positive for *c-fos* in ACKR4^−/−^ suggests an increase in circulating *CCL21* and a consequent increased disease severity. Thus, our study suggests that blockade of *CCL21* in the bladder may have similar disease-modifying effects in BPS patients.

## 5 Conclusions

We have used medium-throughput quantitative polymerase chain reaction analysis to examine genes’ differences in BPS and control biopsies and correlated gene expression to disease phenotype. In addition, we highlighted the role of *CCL21* and *FGF7* in BPS pain in rats and demonstrated an increase in pain processing in mouse knockouts for the *CCL21* atypical receptor. Our results support the potential for the molecular evaluation and manipulation of these inflammatory mediators in the bladder as a possible new treatment strategy for BPS.

  ***Author contributions:*** Ifeoma Offiah had full access to all the data in the study and takes responsibility for the integrity of the data and the accuracy of the data analysis.  

*Study concept and design:* Offiah, O’Reilly, McMahon.

*Acquisition of data:* Offiah, O’Reilly, O'Sullivan, Cartwright, Khullar.

*Analysis and interpretation of data:* Offiah, Didangelos, Dawes, Williams, Chessell, Pallas, Graham, McMahon, O’Reilly.

*Drafting of the manuscript:* Offiah, Dawes, Didangelos, McMahon.

*Critical revision of the manuscript for important intellectual content:* McMahon, Williams, Chessell, Pallas, Graham.

*Statistical analysis:* Offiah, Didangelos.

*Obtaining funding:* McMahon, O’Reilly, Khullar.

*Administrative, technical, or material support:* None.

*Supervision:* McMahon.

*Other (specify):* None.  

***Financial disclosures:*** Ifeoma Offiah certifies that all conflicts of interest, including specific financial interests and relationships and affiliations relevant to the subject matter or materials discussed in the manuscript (eg, employment/affiliation, grants or funding, consultancies, honoraria, stock ownership or options, expert testimony, royalties, or patents filed, received, or pending), are the following: None.  

***Funding/Support and role of the sponsor:*** Funding for the PhD was received from MedImmune. The International Urogynaecological Association Research Grant and MedImmune, a subsidiary of AstraZeneca, provided financial support for this project. The MRC funded the replication study from Imperial College London. The sponsors were involved in the design and conduct of the study, the interpretation of the data, and approval of the manuscript.  

***Acknowledgments:*** The authors acknowledge Dr Orfhlaith O'Sullivan and Dr Elwaleed Babikar, who assisted with the collection of bladder biopsies in the operating room. We would also like to acknowledge the staff of the Urodynamic Department of the Cork University Maternity Hospital, most notably S/M Elaine Dilloughery and S/M Eleanor O’Connell, who assisted in the recruitment of patients. We would like to acknowledge Larissa Franklin for sample handling. Special thanks to Dr Barbara Häenzi for proofreading the paper.

## References

[1] van de Merwe J.P., Nordling J., Bouchelouche P. (2008). Diagnostic criteria, classification, and nomenclature for painful bladder syndrome/interstitial cystitis: an ESSIC proposal. Eur Urol.

[2] Abrams P., Cardozo L., Fall M. (2002). The standardisation of terminology of lower urinary tract function: report from the Standardisation Sub-committee of the International Continence Society. Am J Obstet Gynecol.

[3] Jones C.A., Nyberg L. (1997). Epidemiology of interstitial cystitis. Urology.

[4] Parsons C.L. (2007). The role of the urinary epithelium in the pathogenesis of interstitial cystitis/prostatitis/urethritis. Urology.

[5] Harrington D.S., Fall M., Johansson S.L. (1990). Interstitial cystitis: bladder mucosa lymphocyte immunophenotyping and peripheral blood flow cytometry analysis. J Urol.

[6] Bartus K., James N.D., Bosch K.D. (2014). Large-scale chondroitin sulfate digestion with chondroitinase gene therapy leads to reduced pathology and modulates macrophage phenotype following spinal cord contusion injury. J Neurosci.

[7] Cafferty W.B., Bradbury E.J., Lidierth M. (2008). Chondroitinase ABC-mediated plasticity of spinal sensory function. J Neurosci.

[8] Schmittgen T.D., Livak K.J. (2008). Analyzing real-time PCR data by the comparative C(T) method. Nat Protoc.

[9] Perkins J.R., Dawes J.M., McMahon S.B., Bennett D.L., Orengo C., Kohl M. (2012). ReadqPCR and NormqPCR: R packages for the reading, quality checking and normalisation of RT-qPCR quantification cycle (Cq) data. BMC Genomics.

[10] Stegemann C., Drozdov I., Humphries J. (2011). Comparative lipidomics profiling of human atherosclerotic plaques. Circ Cardiovasc Genet.

[11] Lee U.J., Ackerman A.L., Wu A. (2015). Chronic psychological stress in high-anxiety rats induces sustained bladder hyperalgesia. Physiol Behav.

[12] Chaplan S.R., Bach F.W., Pogrel J.W., Chung J.M., Yaksh T.L. (1994). Quantitative assessment of tactile allodynia in the rat paw. J Neuroscience Methods.

[13] Comerford I., Nibbs R.J., Litchfield W. (2010). The atypical chemokine receptor CCX-CKR scavenges homeostatic chemokines in circulation and tissues and suppresses Th17 responses. Blood.

[14] McMahon S.B., Abel C. (1987). A model for the study of visceral pain states: chronic inflammation of the chronic decerebrate rat urinary bladder by irritant chemicals. Pain.

[15] Keay S.K., Birder L.A., Chai T.C. (2014). Evidence for bladder urothelial pathophysiology in functional bladder disorders. Biomed Res Int.

[16] Logadottir Y., Delbro D., Lindholm C., Fall M., Peeker R. (2014). Inflammation characteristics in bladder pain syndrome ESSIC type 3C/classic interstitial cystitis. Int J Urol.

[17] Colaco M., Koslov D.S., Keys T. (2014). Correlation of gene expression with bladder capacity in interstitial cystitis/bladder pain syndrome. J Urol.

[18] Gamper M., Viereck V., Eberhard J. (2013). Local immune response in bladder pain syndrome/interstitial cystitis ESSIC type 3C. Int Urogynecol J.

[19] O’Reilly B.A., Kosaka A.H., Chang T.K., Ford A.P., Popert R., McMahon S.B. (2001). A quantitative analysis of purinoceptor expression in the bladders of patients with symptomatic outlet obstruction. BJU Int.

[20] Jiang Y.H., Peng C.H., Liu H.T., Kuo H.C. (2013). Increased pro-inflammatory cytokines, C-reactive protein and nerve growth factor expressions in serum of patients with interstitial cystitis/bladder pain syndrome. PLoS One.

[21] Liu H.T., Kuo H.C. (2012). Increased urine and serum nerve growth factor levels in interstitial cystitis suggest chronic inflammation is involved in the pathogenesis of disease. PLoS One.

[22] Yen T.T., Thao D.T., Thuoc T.L. (2014). An overview on keratinocyte growth factor: from the molecular properties to clinical applications. Protein Pept Lett.

[23] Meropol N.J., Somer R.A., Gutheil J. (2003). Randomized phase I trial of recombinant human keratinocyte growth factor plus chemotherapy: potential role as mucosal protectant. J Clin Oncol.

[24] Rosen L.S., Abdi E., Davis I.D. (2006). Palifermin reduces the incidence of oral mucositis in patients with metastatic colorectal cancer treated with fluorouracil-based chemotherapy. J Clin Oncol.

[25] Pickens S.R., Chamberlain N.D., Volin M.V., Pope R.M., Mandelin A.M., Shahrara S. (2011). Characterization of CCL19 and CCL21 in rheumatoid arthritis. Arthritis Rheum.

[26] Biber K., Tsuda M., Tozaki-Saitoh H. (2011). Neuronal CCL21 up-regulates microglia P2X4 expression and initiates neuropathic pain development. EMBO J.

[27] Bonacchi A., Petrai I., Defranco R.M. (2003). The chemokine CCL21 modulates lymphocyte recruitment and fibrosis in chronic hepatitis C. Gastroenterology.

[28] Pierce E.M., Carpenter K., Jakubzick C. (2007). Therapeutic targeting of CC ligand 21 or CC chemokine receptor 7 abrogates pulmonary fibrosis induced by the adoptive transfer of human pulmonary fibroblasts to immunodeficient mice. Am J Pathol.

[29] Lucas B., White A.J., Ulvmar M.H. (2015). CCRL1/ACKR4 is expressed in key thymic microenvironments but is dispensable for T lymphopoiesis at steady state in adult mice. Eur J Immunol.

