# Supplementary material for: The Expression of Inflammatory Mediators in Bladder Pain Syndrome
Source: Eur Urol. 2016 Aug;70(2):283–90. doi: 10.1016/j.eururo.2016.02.058 (PMC4926725; doi:10.1016/j.eururo.2016.02.058)
Supplement: Supplementary file 1 [file mmc1.docx]

**Supplement 1 – Methods**

All procedures performed on human volunteers were approved by the Cork University Research Ethics Committee. A written informed consent was obtained.

Thirty patients were recruited from the Urogynaecology out patient Department of the Cork University Maternity Hospital; 15 female patients with BPS, and 15 female patients with urodynamic stress incontinence, normal cystoscopic exams and no bladder pain were recruited as controls. There was concordance in the patient age, BMI and risk factor assessment between the disease and control groups (Supplementary Table 1). BPS patients had a higher overall past medical history of UTI than controls. This may be due to misdiagnosis by primary care physicians prior to the diagnosis of BPS by the Urologist or Urogynaecologist. However, all mid-stream urine specimens for all participants prior to study recruitment were normal with no bacterial growth on urine culture.

Studies evaluating BPS gene expression profiles often divide BPS patients in terms of Hunner’s versus non-Hunner’s disease. However, experience and published reports suggests that there is often poor correlation between the patient reported disease phenotype and the severity of findings observed at cystoscopy*.* The ICS/PI Questionnaire however, is representative of the global syndrome experienced by the patients and is used to assess disease burden on patients. We therefore examined the correlation between participant phenotype and gene expression levels using this questionnaire. None of the BPS participants had received BPS treatment for at least 3 months prior to study inclusion.

Urodynamic assessment showed a significant decrease in each urodynamic parameter and volume in BPS patients compared to control subjects (Supplementary Fig. 1A). The controls had urodynamic stress urinary incontinence. All BPS patients included in the study had cystoscopic evidence of disease: glomerulations, petechial haemorrhages or Hunner’s lesions following hydrodistension (Supplementary Fig. 1b). The control participants had no lesions on cystoscopy and had normal compliance with hydrodistension. A systematic approach for cystoscopic evaluation of findings was not adopted. Therefore, we were unable to perform stringent analysis on the cystoscopic data. Three 5-mm bladder biopsies were taken from each patient from above the bladder trigone and snap frozen in liquid nitrogen. Biopsies were taken away from lesion sites from healthy looking bladder wall. There were no complications following bladder biopsy in any of the participants.

*Replication study cohort*

Participants from the primary study were recruited based on cystoscopic evidence of disease, adhering to the International Continence Society disease classification of interstitial cystitis. This we acknowledge was a very stringent method of recruitment. In order to confirm the results of the primary discovery study a replicate analysis was performed on an independent group of 38 participants. The bladder biopsies were acquired from collaboration with Imperial College London. They recruited women attending their Urogynaecology department for various urological complaints such as overactive bladder, stress urinary incontinence, recurrent urinary tract infections and bladder pain. Case definitions for stress and urge incontinence and bladder pain were based on the International Consultation on Incontinence Modular Questionnaire – Female Lower Urinary Tract Symptoms (ICIQ-FLUTS) questionnaire. The BPS patient group was recruited based on a history of bladder pain on the questionnaire, and control subjects were patients with other lower urinary tract disorders, such as stress urinary incontinence or overactive bladder, without pain. Thus the group was divided into 23 patients with bladder pain and 15 controls without pain. This cohort was therefore formed of an array of urological patients with the main distinguishing factor for comparison being bladder pain. This cohort thus represents the less severe classification of the disease and describes the actual symptom complex as determined by the European Society for the Study of IC/BPS. Biopsies were taken under general anaesthesia from the bladder dome away from lesion sites in a similar fashion as in the primary discovery study using the cold cup biopsy technique. No other diseases of the bladder were identified at cystoscopy. Each biopsy was snap frozen and stored at −80ºC in RNase-free vials until further RNA extraction and consequent gene expression analysis as detailed in the methods section.
