# Supplementary material for: The Expression of Inflammatory Mediators in Bladder Pain Syndrome
Source: Eur Urol. 2016 Aug;70(2):283–90. doi: 10.1016/j.eururo.2016.02.058 (PMC4926725; doi:10.1016/j.eururo.2016.02.058)
Supplement: Supplementary file 2 [file mmc2.docx]

**Supplementary Table 1 –** **Patient demographics and** **past medical history for bladder pain syndrome and control participants**

| **Demographics** | **Control n = 15** | **BPS n = 15** |
| --- | --- | --- |
| Age yr, mean ± SD | 51.7 ± 10.6 | 50.9 ± 16.9 |
| Weight Kg, mean ± SD | 68.7 ± 10.2 | 64.9 ± 18.7 |
| % smoker | 13.30% | 6.70% |
| Past Medical History |  |  |
| Depression | 20% | 20% |
| Hypothyroidism | 13% | 26% |
| Recurrent UTI | 0 | 20% |
| Other pain conditions | 20% | 13% |
| Cystoscopic findings |  |  |
| Trabeculations | 0 | 62% |
| Petechiae | 0 | 100% |
| Hunner’s lesions | 0 | 15% |

SD: Standard Deviation, UTI: Urinary Tract Infections.

There is no difference between BPS and control participant demographics. All BPS patients had at least one cystoscopic finding indicative of BPS, while all the controls had normal bladders.
