# Supplementary material for: The Expression of Inflammatory Mediators in Bladder Pain Syndrome
Source: Eur Urol. 2016 Aug;70(2):283–90. doi: 10.1016/j.eururo.2016.02.058 (PMC4926725; doi:10.1016/j.eururo.2016.02.058)
Supplement: Supplementary file 3 [file mmc3.docx]

**Supplementary Fig.** **1 – Urodynamic and cystoscopic assessment**. **A.** Urodynamic findings. Each sensation is noted as a volume during filling. First desire = first sensation of the urge to urinate, normal desire = sensation when the patient would normally urinate, strong desire = sensation at which patient feels the urgent need to urinate. MCC: Maximum cystometric capacity = sensation at which patient would have urge incontinence if urination is deterred, CC: Cystometric capacity = bladder capacity at the end of the filling phase. Statistical analysis was performed using Two-way ANOVA with Bonferroni post-test correction, comparing BPS versus control patients. ** p-value < 0.01, *** p-value < 0.001. **B.** Hunner’s lesions and petechial haemorrhages on cystoscopy, courtesy of the Urogynaecology Theatres of the CUMH.
